# Supplementary material for: Patterns of Intron Gain and Loss in Fungi
Source: PLoS Biol. 2004 Nov 30;2(12):e422. doi: 10.1371/journal.pbio.0020422 (PMC532390; doi:10.1371/journal.pbio.0020422)
Supplement: Table S1 — Also available at http://genes.mit.edu/NielsenEtAl/. (4.3 MB ZIP). [file pbio.0020422.st001.zip › NielsenEtAl/html/46.html]

AN3793.1.NCU07489.1.MG00149.1.FG00852.1


```
 CLUSTAL W (1.82) Multiple Sequence Alignments - Introns Inserted


Sequence 1: MG00149.1	522 aa
Sequence 2: FG00852.1	534 aa
Sequence 3: NCU07489.1	531 aa
Sequence 4: AN3793.1	512 aa
Alignment Length: 548 aa
Number Identitical Residues: 328 aa
Alignment Score (without introns) 14244


MG00149.1 	MGNSSSKEGGGGSSRSGSGADRGSDGLQSYPSFSRSDTKESSRSFR-SLRSKIPGSGRTD
NCU07489.1	MGNSSSKDAGASSKNRGDDD------LKSYPSFSKSDTKDSSRSFR-GLRSKIPGGSKTD
FG00852.1 	MGNNNSSQGGSGSKGSGSAGPDGP--LQSYPSFSRSDTKDSSRSFR-TLGSKIRSSSKSD
AN3793.1  	MGQSHSK-GNSGAGDS----------LQSYPSFSKSDTKESLRSFRGSIRSKIRSS---D
          	**:. *. ....:             *:******:****:* ****. : *** ..   *

MG00149.1 	SPRNSAIVTTNGDYGDKSDAASVKSG-VSRQGRTDSN-LS--------------ATSPTD
NCU07489.1	SPRNSTILS-SEEVAEKMNALSGKNGRPSVHTRPSRRDMSPSPASP-STSSPSSPSLDTA
FG00852.1 	SPRNSKVLS-NGENPVESKSDDRRS---SRHGRSSSSRLSRSELPPLNTAGTDISTSESA
AN3793.1  	SPRAS--TSALNQSDDKSDAGSMKS----VGSRRSSTNLSR-------------PASADS
          	*** *   ::  :   : .: . :.       * .   :*              .:    

MG00149.1 	TIQSPIDEPQLPPSPVHASHKQGAKHDVSAAQASGEVDHVSDQPPGQGGVPNPTAQSQTP
NCU07489.1	TLASPTGEPLPPPSPVHN--ATGGHHDVRAAQESGEVDHISDQPPSGGGGAN---SNQQP
FG00852.1 	LADSAVGEDQPPPSPVHG-NAKGGNHDVSAAQASGEVDHVSDQPPSVNAGAN--AHMQAP
AN3793.1  	SAASPTDAPDPPPSPTMSSSLKRGHQDVAAMKQSGEVDHVSDAPPSAAPPPG---SQAKV
          	   *. .    ****.  :    .::** * : ******:** **.    ..        

MG00149.1 	GQSILVKREDAVNPVRTAGS--PREDG-------VAMSEIKDIDLDDFIKRLLDAAYAGK
NCU07489.1	GQSILVKRENTINPVYDTPNQDPQNEQAVSG---VAMSDIKDIDLDDFIKRLLDAAYAGK
FG00852.1 	GQSILVKRENTINPVNNGPSDESRADGNSN----VAMSEIKDIDLDDFIKRLLDAGYAGK
AN3793.1  	GESILIKREDTSNPILDFIMKTPLHSDASSSSPGMGMGALKSIDLDDMITRLLDAGYSTK
          	*:***:***:: **:     . .  .   ..:..:.*. :*.*****:*.*****.*: *

MG00149.1 	VTKSVCLKNAEIAAICHRARECFLSQPALLELDAPVKIVGDVHGQYTDLIRMFEMCGFPP
NCU07489.1	VTKSVCLKNAEIVAICHRARELFLSQPALLELDAPVKIVGDVHGQYTDLIRMFEMCGFPP
FG00852.1 	VTKSVCLKNAEIVAICQRAREVFLSQPALLELDAPVKVVGDVHGQYTDVIRMFEMCGFPP
AN3793.1  	VTKTVCLKNAEITAICTAARELFLSQPALLELSAPVKIVGDVHGQYTDLIRLFEMCGFPP
          	***:********.***  *** **********.****:**********:**:********

MG00149.1 	NSNFLFLGDYVDRGKQSLETILLLLCYKLKYPENFFLLRGNHECANVTRVYGFYDECKRR
NCU07489.1	NSNYLFLGDYVDRGKQSLETILLLMCYKLKYPENFFLLRGNHECANVTRVYGFYDECKRR
FG00852.1 	NSNYLFLGDYVDRGKQSLETILLLLCYKLKFPENFFLLRGNHECANVTRVYGFYDECKRR
AN3793.1  	VSNYLFLGDYVDRGKQSLETILLLLCYKLKFPENFFLLRGNHECANVTRVYGFYDECKRR
          	 **:********************:*****:*****************************

MG00149.1 	CNVKVWKTFIDTFNTLPIAAIVAGKIFCVHGGLSPALVHMDDIRNIARPTDVPDYGLLND
NCU07489.1	CNVKIWKTFVDTFNCLPIAAIVAGKIFCVHGGLSPALGHMDDIRNIARPTDVPDYGLLND
FG00852.1 	CNVKIWKTFIDCFNTLPIAAIVAGKIFCVHGGLSPALVHMDDIRNIARPTDVPDYGLLND
AN3793.1  	CNIKIWKTFVDTFNCLPIAATVAGKIFCVHGGLSPSLSHMDDIRNIARPTDVPDYGLLND
          	**:*:****:* ** ***** **************:* **********************

MG00149.1 	LLWSDPADMEQDWEANERGVSYCFGKKVITEFLAQHDFDLVCRAHMVVEDGYEFFNDRVL
NCU07489.1	LLWSDPADMDQDWEANERGVSYCFGKKVISDFLATHDFDLVCRAHMVVEDGYEFYTDRIL
FG00852.1 	LLWSDPADMEQDWEANERGVSYCFGKRVITEFLAVHDFDLICRAHMVVEDGYEFFNDRVL
AN3793.1  	LLWSDPADMDEDWEPNERGVSYCFNKNVIMNFLQRHDFDLVCRAHMVVEDGYEFYQDRIL
          	*********::***.*********.*.** :**  *****:*************: **:*

MG00149.1 	VTVFSAPN0YCGEFDNWGAVMSVSSELLCSFELLKPLDSNALKSHIKKGRNKRQHMLNSP
NCU07489.1	VTIFSAPN0YCGEFDNWGAVMAVSTELLCSFELLKPLDSAALKTHIKKGRHRRNNMLNSP
FG00852.1 	VTVFSAPN0YCGEFDNWGAVMSVSAELLCSFELLKPLDSSALKSHIKKSRNKRQHMLNSP
AN3793.1  	VTVFSAPN0YCGEFDNWGAVMSVSGELLCSFELLKPLDSAALKSHIKKGRKERNSLINSP
          	**:***** ************:** ************** ***:****.*:.*: ::***

MG00149.1 	0PASVYPQSV
NCU07489.1	0PAHFNPQSV
FG00852.1 	0PAMIQPQSM
AN3793.1  	0PAVVSAQSF
          	 ** . .**.
```
